# TRP Family Genes Are Differently Expressed and Correlated with Immune Response in Glioma

**DOI:** 10.3390/brainsci12050662

**Published:** 2022-05-19

**Authors:** Chaoyou Fang, Houshi Xu, Yibo Liu, Chenkai Huang, Xiaoyu Wang, Zeyu Zhang, Yuanzhi Xu, Ling Yuan, Anke Zhang, Anwen Shao, Meiqing Lou

**Affiliations:** 1 Department of Neurosurgery, Shanghai General Hospital, School of Medicine, Shanghai Jiao Tong University, Shanghai 200080, China; chaoyouf@163.com (C.F.); houshi@sjtu.edu.cn (H.X.); yuanling093@163.com (L.Y.); 2 Department of Neurosurgery, Second Affiliated Hospital, School of Medicine, Zhejiang University, Hangzhou 310009, China; 17853735042@163.com (Y.L.); 21818274@zju.edu.cn (X.W.); 12018507@zju.edu.cn (Z.Z.); theanke@163.com (A.Z.); shaoanwen@zju.edu.cn (A.S.); 3 Department of Molecular and Cellular Biology, University of California, Berkeley, CA 94720, USA; aly1573592486@aliyun.com; 4 Department of Neurosurgery, Huashan Hospital, Shanghai Medical College, Fudan University, Shanghai 200040, China; dr.yuanzhi.xu@gmail.com

**Keywords:** TRP family genes, glioma, prognosis, immunotherapy, bioinformatics analysis

## Abstract

(1) Background: glioma is the most prevalent primary tumor of the human central nervous system and accompanies extremely poor prognosis in patients. The transient receptor potential (TRP) channels family consists of six different families, which are closely associated with cancer cell proliferation, differentiation, migration, and invasion. TRP family genes play an essential role in the development of tumors. Nevertheless, the function of these genes in gliomas is not fully understood. (2) Methods: we analyze the gene expression data of 28 TRP family genes in glioma patients through bioinformatic analysis. (3) Results: the study showed the aberrations of TRP family genes were correlated to prognosis in glioma. Then, we set enrichment analysis and selected 10 hub genes that may play an important role in glioma. Meanwhile, the expression of 10 hub genes was further established according to different grades, survival time, IDH mutation status, and 1p/19q codeletion status. We found that TRPC1, TRPC3, TRPC4, TRPC5, TRPC6, MCOLN1, MCOLN2, and MCOLN3 were significantly correlated to the prognosis in glioma patients. Furthermore, we illustrated that the expression of hub genes was associated with immune activation and immunoregulators (immunoinhibitors, immunostimulators, and MHC molecules) in glioma. (4) Conclusions: we proved that TRP family genes are promising immunotherapeutic targets and potential clinical biomarkers in patients with glioma.

## 1. Introduction

As the most prevalent subgroup of primary intracranial tumors, glioma accounts for 24.5% of all primary brain tumors and 80.9% of malignant primary brain tumors [1,2]. According to the WHO Classification of CNS tumors, the diagnostic categories can partly be defined by genotype, and this was the first study to define glioma according to the presence or absence of IDH mutation and 1p/19q codeletion [3,4]. Currently, the treatment for glioma holds three predominant tenants: surgical resection, external beam radiation therapy, and chemotherapy. However, even for the most promising treatment, the effect of immunotherapy still falls far short of our expectations. Systemic medications cannot reach a therapeutic concentration inside solid tumor tissues and are accompanied by significant systemic side effects [5,6].

Currently, targeted therapy has become an important method in individualized treatment for glioma. Previous studies revealed that gliomas have a high immune infiltration rate [7,8]. Immune checkpoints are costimulators or cosuppressors required to produce an immune response [9]. Affecting immune checkpoints has achieved a breakthrough in the treatment of glioma. For instance, immune-checkpoint-blocking therapy is effective in a GBM mouse model, which suggests that immune checkpoints may be a potential clinical strategy for glioma [10]. Transient receptor potential (TRP) channels are widely expressed on the plasma membrane in numerous types of cells. It consists of six different families, including TRPC (TRP canonical), TRPV (TRP vanilloid), TRPM (TRP melastatin), TRPML (TRP mucolipin), TRPP (TRP polycystin), and TRPA (TRP ankyrin) [11]. TRP channels are cation channels, which are associated with cancer [12]. The TRP family genes related to various diseases and their role in cancer are of particular concern, such as breast cancer, prostate cancer, melanoma, and tract cancers [12,13,14]. mRNA levels for PARP1, PARP2, and TRPM2 genes are deregulated in acute myelogenous leukemia cells [15]. The plasma membrane of triple-negative breast cancer (TNBC) Cells MDA-MB-231 were found to overexpress TRPC3 [16]. Recently, the functions of TRP family genes in glioma have been of great importance. Evidence has shown that TRP family genes are associated with tumor proliferation, the cell cycle, apoptosis, resistance to treatment, and migration [17]. In glioma, TRP family genes could play either an oncogenic role or suppressive role in tumors. In a previous study, TRP family genes were demonstrated to play a crucial role in tumor death and therapy in glioma. For instance, TPRC3 suppresses cell proliferation and promotes cell death in glioma. A previous study revealed that TRPV2 can decrease cell proliferation in hGBM cells and GSCs and induce apoptosis and autophagy in tumor cells [18]. TRPML2 also plays a crucial role in the progression of glioma [17]. Although TRP family genes have an effect on tumorigenesis in a variety of tumors, the underlying mechanism of TRP family genes in glioma is still not very clear. TRPC1 channels may affect glioma cell division mainly by regulating calcium signaling during cytokinesis [19]. TRPC6 induces a sustained elevation of intracellular calcium in conjunction with activation of the calcineurin-nuclear factor of the activated T-cells (NFAT) pathway. By inhibiting the calcineurin-NFAT pathway, the development of malignant GBM phenotypes under hypoxia is significantly reduced [20].

The relationship between TRP family genes and inflammatory markers and inhibitory molecules have been proved in several studies. A fatty acid amide, palmitoylethanolamide (PEA), has been proven to have anti-inflammatory and analgesic properties by activating TRPV1 [21,22]. Bang et al. found that dimethylallyl pyrophosphate (DMAPP) induces acute inflammation by activating TRPV4 [23]. According to Zhao et al., Cathepsin S (Cat-S), which is released by antigen-presenting cells during inflammation, activates PAR2 and TRPV4 to cause inflammation [24]. Inflammation has been well established as a significant factor in cancer development [25]. The use of inflammation markers in cancer immunotherapy has been proven promising [26].

In our present study, we used data from The Tumor Genome Atlas (TCGA) database to analyze the expression of TRP family genes in glioma and their relationship with clinicopathological characteristics. Moreover, we also performed bioinformatic analysis to explore the potential functions of the TRP family genes. The analysis showed that TRP family genes may be potential immunotherapeutic targets and novel clinical biomarkers for patients with glioma.

## 2. Materials and Methods

### 2.1. Genetic Alteration of TRP Family Genes in Glioma

CBioPortal (http://www.cbioportal.org/) (accessed on 7 November 2021) is a free web resource for visualizing and analyzing cancer genomics data in a multidimensional manner and was used to determine the correlation between aberrations of TRP family genes and survival time in glioma [27]. Additionally, the correlation was measured by KM diagrams. We selected sixteen cancers (including breast cancer, colon cancer, cholangiocarcinoma, glioma, kidney cancer, liver cancer, uterus corpus endometrial carcinoma, esophageal cancer, lung cancer, pancreatic cancer, stomach cancer, prostate cancer, head and neck cancer, thyroid cancer, skin cancer, and bladder cancer) that are more common and important in human and more abundant in the database. We included all relevant tumor samples in the database for analysis, including IDH1 wild-type and mutated tumors, 1p/19q codeleted tumors and those without this alteration, and also all WHO grades.

### 2.2. Enrichment Analysis and Hub Genes

To detect which sites and pathways the TRP family genes may act on, an enrichment analysis was conducted using GO and KEGG. By using the Bioinformatics database, GO and KEGG enrichment results were visualized using bubble diagrams. Diagrams of GO enrichment analysis included three parts—a biological process (BP) section, a cellular component (CC) section, and a molecular function (MF) section [28]. KEGG, a utility database resource, was used to understand advanced functions and biological systems [29]. In both analyses, GO enrichment and KEGG enrichment were calculated using *p* < 0.05 as the level of significance.

The hub genes were excavated by the STRING database (https://cn.string-db.org/) (accessed on 11 November 2021) and made by CytoHubba plug-in in Cytoscape software. By inputting the 28 TRP family genes in the STRING database, we obtained the data that can be made into a final figure in Cytoscape software. Interaction scores (median confidence) of 0.4 were considered cut-off values. The Cytoscape software was used to establish a network scaffold. Additionally, the CytoHubba plug-in in Cytoscape software can detect and locate the top 10 of the relevant TRP family genes by using the maximal clique centrality (MCC) and mark them in red (high correlation) or yellow (low correlation) colors based on their correlation with glioma.

### 2.3. Validation of Hub Genes

To validate the expression level of hub genes in different grades of HGG vs. LGG, IDH mutation status vs. IDH wildtype status, and 1p/19q codeletion status vs. 1p/19q non-deletion status, CGGA database (http://www.cgga.org.cn/) (accessed on 15 November 2021) was used for analysis [30]. Human Protein Atlas (HPA) database (The Human Protein Atlas), which contains immunohistochemistry-based expression data for over 20 types of cancers, was used to identify the protein expression levels of TRPC1, TRPC6, TRPM7, MCOLN1, MCOLN2, and MCOLN3 in HGG and LGG tissues [31].

### 2.4. Survival Analysis of Hub Genes

The correlation between overall survival (OS) time and hub genes expressed, in addition to disease-free survival (DFS) time and hub genes expression was assessed by the Kaplan–Meier (KM) survival analysis in the GEPIA database (http://gepia.cancer-pku.cn/) (accessed on 18 November 2021) in glioma patients. Additionally, the standard for gene expression analysis was the lower and upper 50% of gene expression, in which standard patients of glioma were categorized into 2 groups. Log-rank was regarded as significant when *p* < 0.05.

### 2.5. Immune Response Prediction

By retrieving literature, we summarized immune cells, immune cell markers, and immune checkpoint molecules, which might be related to glioma [32,33,34]. Using the TIMER2.0 database (http://timer.cistrome.org/) (accessed on 21 November 2021) [35], we detected the relationship between hub genes and the expression levels of immune cell infiltration (including purity, B cell, CD8+ T Cell, CD4+ T Cell, macrophage, neutrophil, dendritic cell), biomarkers (EGFR, EGFL7, CIC, BRAF, H3F3A, HIST1H3B, ATRX, DAXX, PDGFRA, TERT, CHI3L1, MYC, PTEN, S100A8, S100A9, AHSG, SOCS3, NPM1, FTL, BIRC5, GFAP, CXCR4, CTSD, LDHA, TP53, IL13RA2, PROM1, CD44), immune cell markers (CD8A, CD8B, CD3D, CD79A, CD86, CSF1R, CCL2, CD68, IL10, NOS2, IRF5, PTGS2, CD163, VSIG4, MS4A4A, CEACAM8, KIR2DL3, KIR2DL4, KIR3DL1, KIR3DL2, CD1C, NRP1, STAT1, IFNG, STAT3, IL17A, FOXP3, CCR8, STAT5B, TGFB1, PDCD1, GZMB, CD3E, CD2, CD19, KIR3DL3, KIR2DS4, HLA-DPB1, ITGAM, CCR7, KIR2DL1, STAT6, STAT5A, IL13BCL6, ITGAX, TBX21, STAT4, CTLA4, LAG3, HAVCR2, HLA-DQB1, HLA-DRA, HLA-DPA1, TNF, GATA3, IL21) and immune checkpoint molecules (IDO1, LAG3, CD80, PDCD1LG2, CD86, PDCD1, LAIR1, IDO2, CD276, CD40, CD274, HAVCR2, CD28, CD48, VTCN1, CD160, CD44, TNFSF18, CD200R1, TNFSF4, CD200, NRP1, CTLA4, TNFRSF9, COS, TNFRSF8, TNFSF15, TNFRSF14, CD27,BTLA, LGALS9, TMIGD2, TNFRSF25, CD40LG, ADORA2A, TNFRSF4, TNFSF14, HHLA2, CD244, TNFRSF18, BTNL2, C10ORF54, TIGIT, CD70, TNFSF9, ICOSLG, KIR3DL1) in glioma. Subsequently, we selected the module of Gene_Corr in TIMER2.0, inputted the target genes, and then downloaded the correlated data. Using the TBtools application, after we selected the heatmap module from graphics, inputted the selected data, and modified some parameters, the final figures were obtained.

The correlations between the expression level of hub genes and immunoregulators, which contains immunoinhibitors, inmunostimulators, and MHC molecules, were calculated by the TISIDB database (http://cis.hku.hk/TISIDB/) (accessed on 22 November 2021) [36].

### 2.6. Statistical Data

*p*-Values of less than 0.05 were considered statistically significant in all the analyses, except for those specifically mentioned.

## 3. Results

### 3.1. Aberrations in TRP Family Genes Are Correlated with Prognosis in Human Cancers

**Figure 1:**
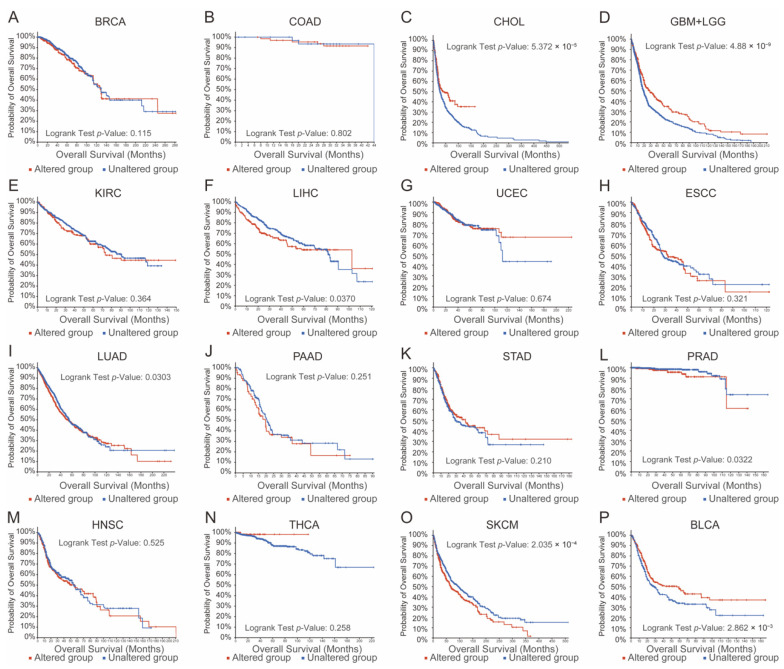
Human cancer prognosis was related to aberrations of genes of the TRP family genes. (**A**–**P**) The relationship between TRPs family genes aberrations and overall survival time in patients with breast cancer, colon cancer, cholangiocarcinoma, glioma, kidney cancer, liver cancer, uterus corpus endometrial carcinoma, esophageal cancer, lung cancer, pancreatic cancer, stomach cancer, prostate cancer, head and neck cancer, thyroid cancer, skin cancer, and bladder cancer.

Using the cBioPortal database, we analyzed the correlation between TRP family aberrations and overall survival (OS) time to evaluate the functional importance of the TRP family in human cancers. As presented in Figure 1, aberrations in the TRP family in the genome were remarkably correlated with shorter OS time in patients with prostate cancer, lung cancer, and skin cancer; however, they were remarkably correlated with better prognosis in patients with cholangiocarcinoma, glioma, bladder cancer, and liver cancer. A significant correlation between the TRP family and survival time was not found in the remaining human cancers. As seen in Figure 1, we evaluated the prognostic usefulness of TRP family genes by pan-cancer analysis. Additionally, we found that aberrations of TRP family genes were associated with a better prognosis among glioma patients.

### 3.2. GO and KEGG Enrichment Analysis and Hub Genes of the TRP Family

**Figure 2:**
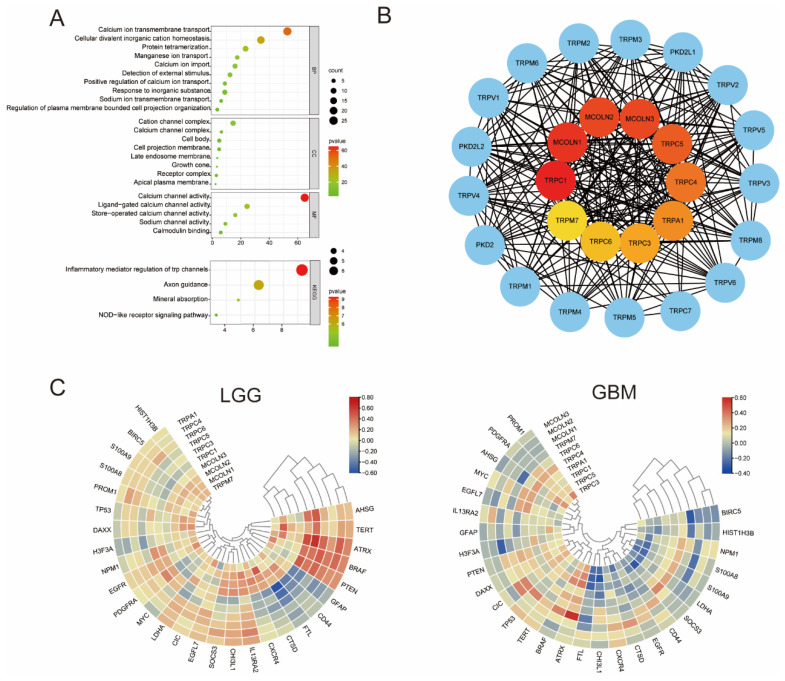
GO and KEGG enrichment analysis and hub genes of the TRP family in glioma. (**A**) The bubble plot shows the most enriched GO and KEGG pathways of target genes. Three factors were considered in the GO enrichment analysis, including BP, CC, and MF. Additionally, the most significant KEGG pathways involved inflammatory and their signaling pathways. (**B**) By using CytoHubba based on the MCC algorithm, the top 10 of the hub genes were identified in the center of the diagram. Additionally, the importance of genes was represented by different colors; red to yellow represent the decreasing importance of the hub genes, while blue was excluded from hub genes. (**C**) Heatmap representing the correlations between hub genes of TRP family and 28 biomarkers in glioma.

To understand downstream pathways of TRP family genes in gliomas, GO and KEGG enrichment analyses were conducted using bioinformatics databases to analyze the potential biological functions of TRP family genes. As shown in Figure 2A, the most significant GO-enriched terms were involved in calcium ion transmembrane transport, cellular divalent inorganic cation homeostasis, and protein tetramerization (BP); cation channel complex, calcium channel complex, and cell body (CC); calcium channel activity, ligand-gated calcium channel activity, and store-operated calcium channel activity (MF). KEGG-enriched terms were mainly involved in the process of inflammatory mediator regulation of TRP channels, axon guidance, and mineral absorption (Figure 2A).

After the potential interaction among these genes was investigated, the CytoHubba plug-in in Cytoscape software was used to reveal the relationship among hub genes. The top 10 hub genes identified included TRPC1, MCOLN1, MCOLN2, MCOLN3, TRPC5, TRPC4, TRPA1, TRPC3, TRPC6, and TRPM7 (Figure 2B). By using the TIMER 2.0 database and TBtools software, the correlation between hub genes and tumor malignancy-associated genes in glioma was determined. As shown in Figure 2C, a majority of hub genes were positively correlated with tumor malignancy.

### 3.3. Amplification, Mutation, and Deletion of Hub Genes in Glioma

**Figure 3:**
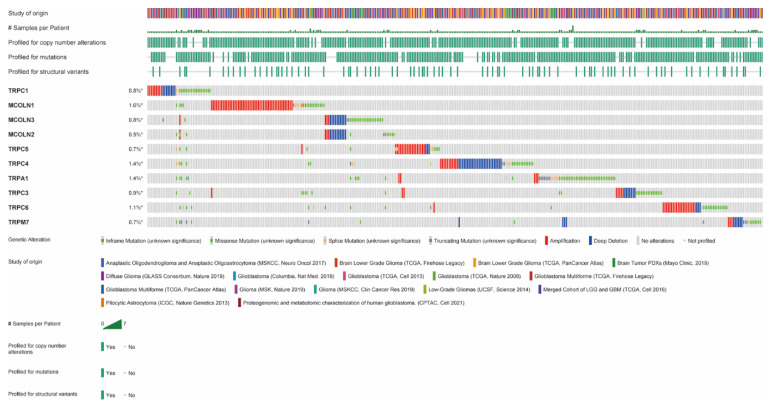
Amplification, deletion, and mutation of hub genes of TRPs family in glioma. Genetic variations in hub genes of the TRPs family were detected using the cBioportal database.

The different functions of hub genes have been involved in several cancers in previous studies, such as pancreatic cancer [37], bladder cancer [38], esophageal squamous cell carcinoma [39], and non-small cell lung carcinoma [40], especially in glioma [41]. The purpose of this study was to explore the functions of TRP family genes in gliomas. CBioPortal was used to identify genetic variations in hub genes in 6853 cases. Among the 10 hub genes, different levels of genetic change were found, including TRPC1, MCOLN1, MCOLN2, MCOLN3, TRPC5, TRPC4, TRPA1, TRPC3, TRPC6, and TRPM7 (Figure 3). The results revealed that all hub genes were amplified, deleted, and mutated in glioma, among which MCOLN1 displayed the highest incidence rate (1.8%).

### 3.4. Expression Difference of Hub Genes in Different Grades of Glioma

**Figure 4:**
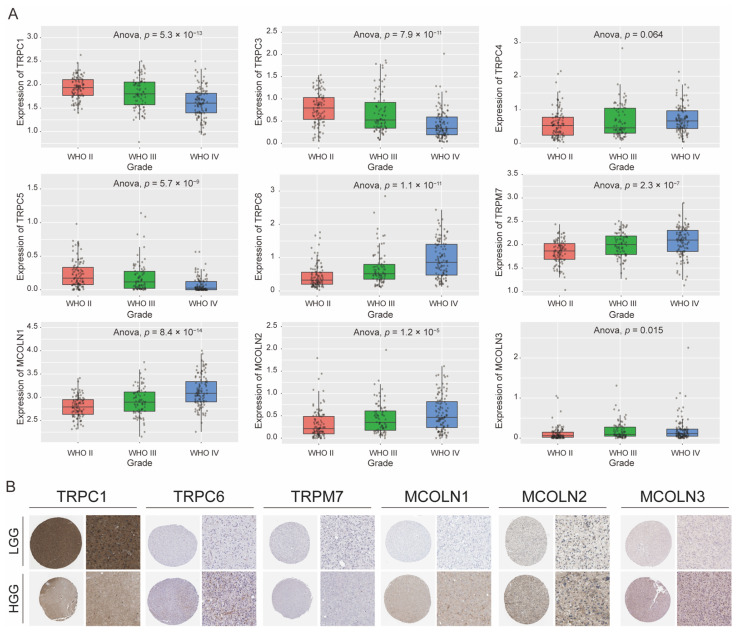
Expression of hub genes in the grade of glioma. (**A**). Validation of hub gene expression levels of TRPC1, MCOLN1, MCOLN2, MCOLN3, TRPC5, TRPC4, TRPC3, TRPC6, and TRPM7 in different grades of glioma using CGGA database. (**B**). Representative immunohistochemistry images of TRPC1, TRPC6, TRPM7, MCOLN1, MCOLN2, and MCOLN3 in HGG and LGG tissues derived from the HAP database. (HGG, high-grade glioma; LGG, low-grade glioma; HAP, Human Protein Atlas).

The results from the GCCA database revealed that the gene expression levels of TRPC1, MCOLN1, MCOLN2, MCOLN3, TRPC5, TRPC4, TRPC3, TRPC6, and TRPM7 in different grades were significantly different. The gene expression levels of TRPC1, TRPC3, and TRPC5 were significantly higher in low-grade glioma (LGG), while the levels of TRPC4, TRPC6, TRPM7, MCOLN1, MCOLN2, and MCOLN3 were significantly higher in high-grade glioma (HGG). All of these results showed that high expression levels of TRPC4, TRPC6, TRPM7, MCOLN1, MCOLN2, and MCOLN3 may correlate with a worse prognosis, while higher expression of TRPC1, TRPC3, and TRPC5 correlates with a better prognosis. However, the expression of TRPC4 in glioma presented no statistical significance (Figure 4A).

By inputting the hub genes as target genes into the HPA database, available representative immunohistochemistry images of TRPC1, TRPC6, TRPM7, MCOLN1, MCOLN2, and MCOLN3 were outputted. As shown in Figure 4B, compared with HGG tissues, the expression of TRPC1 was significantly higher in LGG tissues, while the expression of TRPC6, MCOLN1, MCOLN2, and MCOLN3 was relatively low, and these results are consistent with the above tendency.

### 3.5. Survival Analysis of the Hub Genes in Glioma

**Figure 5:**
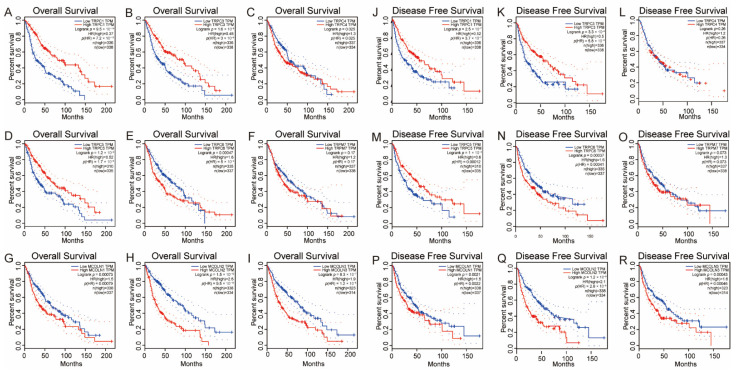
The dysregulation of hub genes of the TRPs family was correlated to OS and DFS time in glioma. (**A**–**I**) higher expression levels of TRPC1 (**A**), TRPC3 (**B**), and TRPC5 (**D**) were significantly associated with better OS time in glioma. Additionally, higher levels of TRPC4 (**C**), TRPC6 (**E**), MCOLN1 (**G**), MCOLN2 (**H**), and MCOLN3 (**I**) were significantly associated with worse OS time in glioma. (**J**–**R**) Similarly, higher levels of TRPC1 (**J**), TRPC3 (**K**), and TRPC5 (**M**) were significantly associated with better DFS time in glioma. Additionally, higher levels of TRPC6 (**N**), MCOLN1 (**P**), MCOLN2 (**Q**), and MCOLN3.

To investigate the prognostic value of the above genes, we performed an analysis of OS and DFS. According to the results, we observed that analysis of the filtered hub genes through the above steps was further conducted by survival plots in the GEPIA database. As demonstrated in Figure 5, higher levels of TRPC1, TRPC3, and TRPC5 were significantly associated with longer OS time in glioma. Higher levels of TRPC4, TRPC6, MCOLN1, MCOLN2, and MCOLN3 were significantly associated with shorter OS times in glioma. Favorable DFS was significantly correlated with higher levels of TRPC1, TRPC3, and TRPC5 but significantly associated with lower levels of TRPC6, MCOLN1, MCOLN2, and MCOLN3. These findings were consistent with the obtained result of the expression profile of hub genes in the grade of glioma, which means that follow-up studies of TRPC1, MCOLN1, MCOLN2, MCOLN3, TRPC5, TRPC4, TRPC3, and TRPC6 will make more sense.

### 3.6. Expression Difference of Hub Genes in IDH Status, 1p/19q Codeletion Status of Glioma

**Figure 6:**
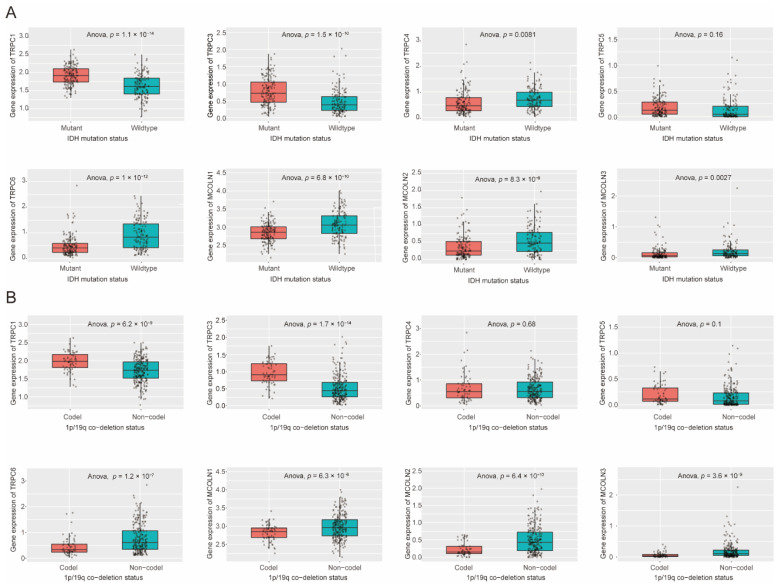
Expression of hub genes in IDH mutation status and 1p/19q codeletion status of glioma. (**A**)**.** Validation of hub gene expression levels of TRPC1, MCOLN1, MCOLN2, MCOLN3, TRPC5, TRPC4, TRPC3, and TRPC6 in different IDH mutations status of glioma using CGGA database. (**B**). Validation of hub gene expression levels of TRPC1, MCOLN1, MCOLN2, MCOLN3, TRPC5, TRPC4, TRPC3, and TRPC6 in different 1p/19q codeletion status of glioma using CGGA database. The bottom and top of the boxes are the 25th and 75th percentiles, interquartile range. The statistical difference between the two subgroups was compared through the Kruskal–Wallis test, respectively.

By using the CGGA database, the expression levels of TRPC1, TRPC3, TRPC4, TRPC5, TRPC6, MCOLN1, MCOLN2, and MCOLN3 with IDH mutation status and 1p/19q codeletion status were compared. The expression levels of TRPC1 and TRPC3 were significantly higher in the 1p/19q codeletion status, while the gene expression levels of TRPC6, MCOLN1, MCOLN2, and MCOLN3 were significantly higher in the 1p/19q non-codeletion status (Figure 6B). IDH, a vital enzyme that participates in several essential metabolic processes, frequently exhibits a mutation status in human malignancies. A previous study indicated that patients with glioma with IDH mutation had a better OS time than those patients with wild-type IDH [42]. Similarly, the codeletion of chromosome status in glioma was associated with longer OS and DFS times, which means that 1p/19q codeletion status in patients with glioma could be used to predict a better prognosis [43]. The results demonstrated that the gene expression levels of TRPC1 and TRPC3 were significantly higher in IDH mutation status, while the gene expression levels of TRPC4, TRPC6, MCOLN1, MCOLN2, and MCOLN3 were significantly higher in IDH wild-type status (Figure 6A). The results were consistent with the survival analysis and expression differences above.

### 3.7. Hub Genes Are Associated with Immune Activation in Glioma

**Figure 7:**
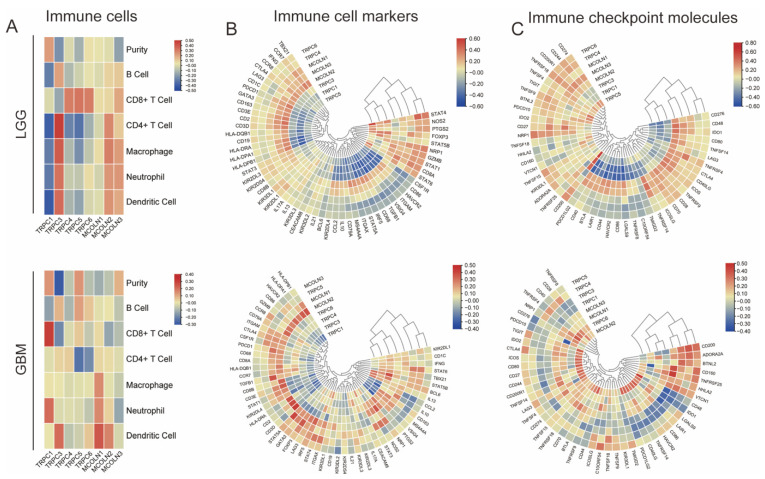
Hub genes are associated with immune activation in glioma. Heatmap represents the correlations between hub genes of TRPs family and 6 infiltrating immune cells (**A**), 56 immune cell markers (**B**), and 47 immune checkpoint molecules (**C**) in GBM and LGG.

By using the TIMER 2.0 database and TBtools software, we revealed the correlation of hub genes with the expression levels of immune cell infiltration, immune cell markers, and immune checkpoint molecules. The results that hub genes correlated with immune cell infiltration, immune cell markers, and immune checkpoint molecules in GBM and LGG are presented in Figure 7. As shown in Figure 7, a majority of hub genes were positively correlated with molecular markers.

### 3.8. Correlations between Hub Genes and the Expression of Immunoregulators in Glioma

**Figure 8:**
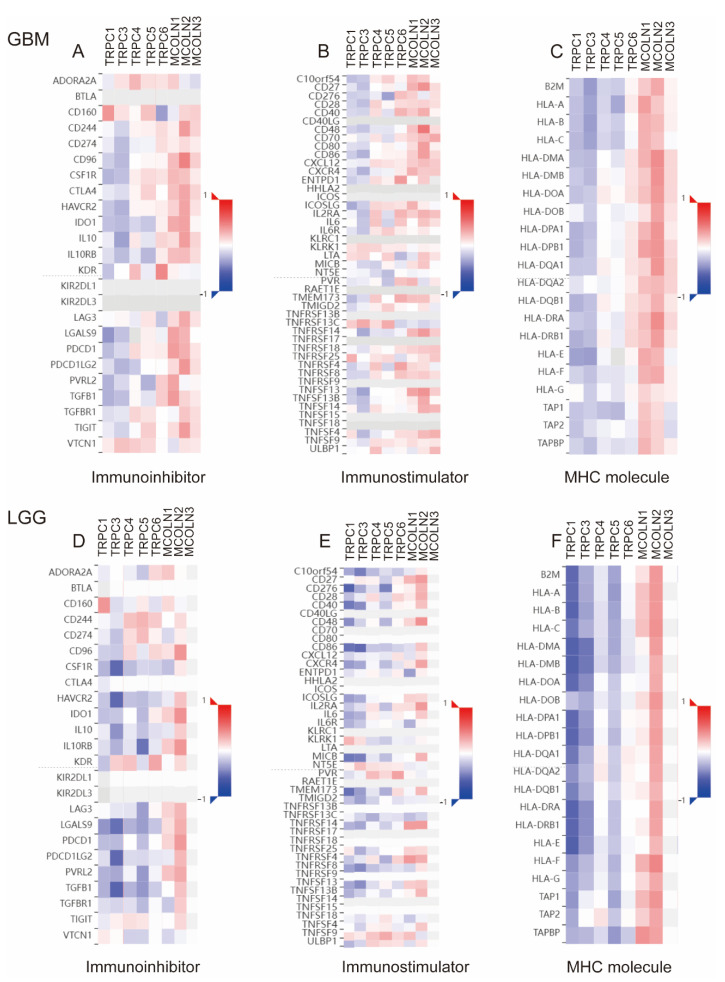
Correlations Between Hub Genes and the Expression of Immunoregulators in Glioma. The correlation between the expression of TRPC1, TRPC3, TRPC4, TRPC5, TRPC6, MCOLN1, MCOLN2, and MCOLN3 and immunoinhibitors (**A**,**D**), immunostimulators (**B**,**E**), and MHC molecules (**C**,**F**) was calculated based on the TISIDB database.

By calculating the correlations between the expression of hub genes and immunoregulators, the effects of hub genes on the tumor immune response were explored (Figure 8). The results indicated that hub genes and the expression of immune regulators exhibited obvious correlations. TRP family genes have been shown to play key roles in inflammatory and immune cells by affecting cytokine production, cell differentiation, and cytotoxicity. In this section, we revealed that the expression of hub genes is correlated with immune activation and immunoregulators in glioma, which indicated that TRP family genes may serve as putative drug targets in glioma.

## 4. Discussion

Several studies have shed light on the role of TRP family genes in glioma in the past few years. In previous studies, the high expression of TRPC1 promoted tumor cell growth and migration through the mechanisms of cytokinesis and chemotaxis, respectively [44,45]. TRPC6 enhances cell proliferation, tumor growth, and angiogenesis, which is essential for glioma [46]. It has been shown that TRPML2 plays an important role in the progression of gliomas through the PI3K/AKT and ERK1/2 pathways [47]. In contrast, TRPM7 suppresses glioma progression through miR-28-5p regulation in GBM cells with subsequent effects on cell proliferation and invasion [48]. TRPML1, mediating autophagic cell death and exerting its cytotoxic effects in glioma, showed a protective effect against glioma progression [49,50]. Nevertheless, there remains a lack of wide analysis of the correlation between TRP family genes and glioma progression. Our study found significant prognostic value for TRP family genes in glioma by analyzing multiple datasets. Additionally, we determined whether TRP family genes affect antitumor immunity.

Our research systematically identified the most promising TRP family genes, which have the potential to be used as potential biomarkers in glioma prognosis and immunotherapy. We found that aberrations in TRP family genes in the genome were significantly correlated with prognosis in human glioma. Then, we performed functional enrichment analysis and found the top 10 hub genes of TRP family genes in glioma. The results showed that TRP family genes are mainly related to calcium ion transmembrane transport, cellular divalent inorganic cation homeostasis, and protein tetramerization in biological processes. In the cellular component category, TRP family genes were mainly enriched in cation channel complexes, calcium channel complexes, and the cell body. In molecular function, TRP family genes mainly correlate to calcium channel activity, ligand-gated calcium channel activity, and store-operated calcium channel activity. In KEGG-enriched terms, inflammatory mediator regulation of TRP channels displayed the highest correlation with TRP family genes. According to the results, we hypothesized that TRP family genes are highly associated with the immune response in glioma. Then, we found the top 10 hub genes in TRP family genes, which could be important genes correlated with immunotherapy and used as biomarkers. A previous study proved that the clinical value of TRPC1, TRPC6, TRPML1, and TRPML2 plays key roles in the diagnosis and prognosis of glioma patients. In our present study, we revealed that several novel TRP family genes may play an important role in glioma progression and be potential therapeutic targets.

Then, we found that aberrations in hub genes in the genome were correlated with prognosis in glioma. As shown in Figure 3, we revealed that all of the hub genes were amplified, deleted, and mutated in glioma, with MCOLN1 exhibiting the highest incidence rate (1.6%). The new studies indicate that MCOLN1/TRPML1 inhibits autophagy by inducing ROS-mediated TP53/p53 pathways, which inhibit cancer metastasis [51]. To verify the correlation between hub gene expression and malignancy in glioma, we investigated the expression of hub genes in glioma with different grades and explored their correlation with the time of OS and DFS. Our results indicated that all hub genes were differentially expressed in glioma grade except TRPA1. It is worth mentioning that although the expression of TRPV4 in different grades of glioma did not meet the inclusion criteria (*p* > 0.05), the difference in expression in OS time was significant. According to the screening criterion, the top 10 hub genes except TRPA1 and TRPM7 were finalized as reference genes for the follow-up study.

Among the eight final identified hub genes, higher expression levels of TRPC1, TRPC3, and TRPC5 were significantly associated with longer OS time in glioma, while higher expression levels of TRPC4, TRPC6, MCOLN1, MCOLN2, MCOLN3 were significantly associated with shorter OS time. In previous reports, TRPC1, TRPC3, TRPC6, TRPML1, and TRPML2 were proven to play crucial roles in glioma progression. Impairing TRPC6 activity in human glioma cells could reduce human xenograft growth in immunocompromised mice [52]. The expression level of TRPML2 was dramatically augmented in high-grade glioblastoma cell lines [47].

To evaluate whether hub genes are related to glioma patient prognosis, we further explored hub gene expression in patients with IDH mutations and 1p/19q codeletions. Previous studies indicated that patients with glioma with IDH mutation or 1p19q codeletion have a more favorable prognosis than patients with wild-type IDH or without 1p/19q codeletion [53,54]. In our present study, the results showed that the expression of TRPC1 and TRPC3 was significantly increased in glioma with IDH mutation status and 1p/19q codeletion status, while the expression of TRPC6, MCOLN1, MCOLN2, and MCOLN3 was significantly decreased. These results indicate that higher expression of TRPC1 and TRPC3 correlates with a better prognosis in patients with glioma, while higher expression of TRPC6, MCOLN1, MCOLN2, and MCOLN3 correlates with a worse prognosis in patients with glioma. The outcomes are consistent with previous results.

In recent years, much progress in immunotherapy has been made, and immunotherapy has promising prospects [55]. Previous studies have shown that immune cells, such as T cells, are closely correlated with tumor progression and clinical prognosis in patients and the response to immunotherapy in both humans and mice [56]. TRP family genes have been shown to play key roles in inflammatory and immune cells by affecting cytokine production, cell differentiation, and cytotoxicity [57]. However, few studies have clarified the relationship between TRP family genes and tumor immunotherapeutic targets. In our present study, we found that the expression of TRP family genes is strongly associated with a variety of cancers, especially glioma.

As shown in our previous study, we found that the TRP gene family was enriched in the inflammatory response. Previous studies have shown that the prognosis of glioma is related to immune cell infiltration, immune cell markers, and immune checkpoint molecules [2,22,58,59,60]. Some immunotherapy approaches, such as immune checkpoint blockade, have been successfully applied in organisms [61]. As a further demonstration of the predictive value of hub genes for immune therapy response in glioma, a heatmap was created to represent the correlation between hub genes and immune markers. According to the correlation between hub genes and immune biomarkers, we can predict the immunotherapy effects. In addition, we further validated the correlation between hub genes and the level of immune cell infiltration in cancer from TISIDB. The results were consistent with the conclusion beforehand.

## 5. Conclusions

In summary, the study identified the dysregulation of eight glioma-associated hub genes of the TRP family through bioinformatics analysis, which is correlated with the prognosis of glioma patients. Thus, it can be used as a prognostic biomarker. Further, we showed that the expression of hub genes is correlated with immune activation and immunoregulators in glioma. Our study indicated that TRP family genes may work as putative drug targets in glioma. We should be aware of several limitations of this study. First of all, the number of validation databases in this article is insufficient. A future study will need to examine other public databases for verification to confirm the results obtained in this study. Secondly, there is a lack of clinical practice in our conclusion, so further clinical trials are needed to confirm whether TRP family genes can be used as new glioma treatment targets. Thirdly, the current study does not yet prove that the hub genes can be used as an independent prognostic factor, which means that further trials are needed. In conclusion, our study identified characteristics of TRP family genes, which can be used to predict prognosis for glioma patients and are associated with immunological response, providing a new potential treatment strategy for glioma patients.

## Data Availability

The original contributions presented in the study are included in the article, further inquiries can be directed to the corresponding authors.
